# Santalol Suppresses Lung Metastasis of Colorectal Cancer Cells by Increasing ROS Levels

**DOI:** 10.3390/antiox15101361

**Published:** 2026-10-21

**Authors:** Sang-Yong Han, Su-Jin Kim, Geun-Ah Jo, Se-Geon Oh, Ji-Ye Kee

**Affiliations:** Department of Oriental Pharmacy, College of Pharmacy, Wonkwang-Oriental Medicines Research Institute, Wonkwang University, 460 Iksandae-ro, Iksan 54538, Jeonbuk, Republic of Korea; hsyangel@wku.ac.kr (S.-Y.H.);

**Keywords:** santalol, colorectal cancer, ROS, mitochondrial dysfunction, apoptosis, lung metastasis

## Abstract

Santalol (STL), a sesquiterpene alcohol extracted from sandalwood oil, has reported antioxidant, anti-inflammatory, and anticancer effects. Nevertheless, the influence of this compound on lung metastasis of colorectal cancer (CRC) cells has not been insufficiently elucidated. In this study, we investigated the anticancer activity of STL in metastatic CRC cells. STL significantly reduced cell viability and clonogenic capacity in a dose-dependent manner. This compound induced mitochondria-mediated apoptosis of CRC cells by regulating caspases and PARP. In addition, it reduced total antioxidant capacity and increased reactive oxygen species (ROS) levels. These results indicate that STL can enhance mitochondrial depolarization and activate the mitochondrial apoptotic pathway. Using an in vivo murine model, we further demonstrated that STL restricted the pulmonary dissemination of CRC cells. Taken together, these findings reveal that STL hinders lung metastasis of CRC cells via the induction of apoptosis and mitochondrial dysfunction driven by intracellular ROS accumulation. Our findings support STL as a promising therapeutic candidate for metastatic CRC.

## 1. Introduction

Colorectal cancer (CRC), which arises in the colon or rectum, has high incidence and mortality rates worldwide. In 2022, CRC was the second most commonly diagnosed cancer in men and the third in women globally; it was also the second leading cause of cancer-related death after lung cancer [1]. Approximately 20–25% of patients with CRC are diagnosed with stage IV (metastatic) disease at initial presentation [2]. Moreover, approximately 10–25% of patients diagnosed with stages I–III of the disease develop metastases after resection of the primary tumor [3]. CRC metastases occur primarily in the liver and lungs [4]. The prognosis of metastatic CRC is particularly poor, with a 5-year survival rate of only 14% when the disease has spread to distant organs [5]. In addition, conventional CRC treatments, such as 5-fluorouracil (5-FU) and oxaliplatin, are associated with various adverse effects [6]. Therefore, new therapies for metastatic CRC are needed.

Apoptosis acts as a critical regulatory mechanism that sustains tissue equilibrium while systematically eliminating damaged or potentially malignant cells [7]. The process exhibits unique structural changes, encompassing cellular shrinkage, chromatin condensation, and the assembly of apoptotic bodies [8]. Mechanistically, apoptosis proceeds through two major signaling pathways: the extrinsic and intrinsic pathways [9]. The extrinsic pathway, frequently designated as the death receptor pathway, is initiated by the interaction between extracellular ligands and transmembrane receptors belonging to the tumor necrosis factor receptor superfamily [10]. This interaction recruits adaptor proteins that activate initiator caspase-8, thereby triggering a downstream executioner caspase cascade [11]. In contrast, the intrinsic pathway is primarily mediated by mitochondria and is activated by intracellular stressors, such as DNA damage or excessive reactive oxygen species (ROS) [12]. These stimuli increase mitochondrial outer-membrane permeability, resulting in the translocation of cytochrome c into the cytoplasm and the ensuing activation of caspase-9 [13].

Both pathways ultimately activate executioner caspases, such as caspase-3, which coordinate the orderly dismantling of the cell [14]. Compared with normal cells, cancer cells exhibit increased metabolic activity and activated tumorigenic signaling, resulting in high basal ROS levels [15]. Although moderate ROS levels can support tumor progression, this inherent oxidative stress makes cancer cells highly vulnerable to further ROS accumulation that exceeds their antioxidant capacity [16]. Excessive ROS accumulation causes irreversible oxidative damage to essential macromolecules, including DNA, proteins, and lipids [17]. This severe oxidative stress increases mitochondrial membrane permeability, releasing apoptotic factors such as cytochrome c into the cytoplasm [18]. Consequently, caspase cascades and downstream signaling pathways are activated, ultimately inducing apoptosis in cancer cells [19]. Therefore, exploiting this vulnerability may provide a promising therapeutic strategy for selectively eliminating cancer cells while minimizing toxicity to normal cells.

*Santalum album* L. (family Santalaceae) is a well-known medicinal herb whose dried heartwood has traditionally been used to regulate Qi, relieve pain, harmonize the stomach, and arrest vomiting [20]. Santalol (STL) is an active compound (consisting predominantly of α- and β-isomers) found in several species of the genus Santalum, including *Santalum album* (sandalwood). STL accounts for approximately 70–90% of the sandalwood oil derived from this plant [21]. Santalol (STL), a primary bioactive sesquiterpene alcohol derived from *Santalum album*, has attracted growing interest in chemoprevention and oncology. Sesquiterpenes are recognized for their distinct pharmacological potential to interfere with oncogenic signaling and intracellular redox homeostasis [22]. Indeed, recent studies have demonstrated that STL exerts potent antitumor activities against various cancers, such as prostate, breast, and skin cancers [23,24,25,26,27], by triggering intrinsic apoptosis, causing cell cycle arrest, and suppressing cell motility. Because CRC metastasis is profoundly driven by oxidative stress perturbations and aberrant survival kinase cascades, we hypothesized that STL could disrupt CRC survival and metastatic colonization. However, its therapeutic utility and underlying molecular mechanisms in CRC metastasis remain largely unexplored. In this study, we investigated the antimetastatic and pro-apoptotic effects of STL on CRC cells both in vitro and in vivo, with a focus on ROS/AMPK-mediated signaling pathways.

## 2. Materials and Methods

### 2.1. Reagents

Santalol (CAS No. 11031-45-1; 85% purity; mixture of α- and β-isomers) was purchased from Biopurify Phytochemicals Ltd. (Chengdu, China). Because the manufacturer does not specify the isomeric ratio, santalol was used as α- and β-isomer mixture in all experiments. DMEM, RPMI 1640, penicillin–streptomycin (10,000 U/mL), and fetal bovine serum (FBS) were purchased from Thermo Fisher Scientific (Waltham, MA, USA). Antibodies against PARP, caspase-3, caspase-9, p53, cytochrome c, Bax, Bcl-2, Bcl-xL, phospho-PI3K, PI3K, phospho-AKT, AKT, phospho-AMPK, AMPK, phospho-JNK, JNK, phospho-p38, and phospho-ERK were purchased from Cell Signaling Technology (Danvers, MA, USA). Antibodies against p38, ERK, and β-actin were purchased from Santa Cruz Biotechnology (Dallas, TX, USA). Crystal violet, 5-fluorouracil (5-FU), and N-acetylcysteine (NAC) were purchased from Sigma-Aldrich (Burlington, MA, USA).

### 2.2. Cell Viability and IC50 Calculation

The murine CRC cell line CT26, the human CRC cell line HCT116, human normal colon fibroblast cell line CCD-18Co, and mucosal mast cell-like rat basophilic leukemia cell line RBL-2H3 were obtained from the Korean Cell Line Bank (Seoul, Republic of Korea). CT26, CCD-18Co, and RBL-2H3 cells were cultured in DMEM, whereas HCT116 cells were cultured in RPMI 1640 in accordance with the supplier’s recommendations. Both media were supplemented with penicillin (100 U/mL), streptomycin (100 µg/mL), and 10% heat-inactivated FBS. All cells were cultured in a CO_2_ incubator at 37 °C in a humidified atmosphere containing 5% CO_2_. We evaluated cell viability with the D-Plus™ CCK Cell Viability Assay Kit (Dongin LS, Seoul, Republic of Korea) according to the manufacturer’s instructions. CT26 cells (5 × 10^3^ cells/well) and HCT116 cells (1 × 10^4^ cells/well) were seeded in 96-well plates and cultured overnight. The cells were then treated with STL (0–40 µM) for 24 h. The culture medium was replaced with a 1:9 mixture of CCK solution and fresh medium, and absorbance was measured at 450 nm using a microplate reader (Molecular Devices, San Jose, CA, USA). The IC50 values were determined by linear interpolation in Microsoft Excel using the two concentrations (X_1_ and X_2_) with corresponding cell viabilities (Y_1_ and Y_2_) that bracketed 50%, according to the following formula: IC50 = X_1_ + (50 − Y_1_)(X_2_ − X_1_)/(Y_2_ − Y_1_). GraphPad Prism software (version 8.0.2; GraphPad Software, San Diego, CA, USA) was used to graph the data.

### 2.3. Colony Formation

CT26 (5 × 10^2^ cells/well) and HCT116 (1 × 10^3^ cells/well) cells were seeded in 12-well culture plates and allowed to adhere overnight. The cells were treated with STL (0–20 μM) and incubated for 7 days. Following the removal of the culture medium, we fixed the colonies with 10% formaldehyde for 15 min, washed them three times with phosphate-buffered saline (PBS), and stained them with 0.1% crystal violet solution for 20 min. After three additional washes with PBS, the colonies were dried and photographed the following day. To quantify relative colony formation, the stained colonies were dissolved in methanol, and absorbance values were quantified employing a microplate reader.

### 2.4. Flow Cytometric Analysis of Apoptosis and Mitochondrial Membrane Potential

Apoptosis and mitochondrial depolarization in CRC cells were assessed by using the Muse^®^ Annexin V & Dead Cell Kit and Muse^®^ MitoPotential Kit, respectively (Luminex, Austin, TX, USA). CT26 (2 × 10^5^ cells/well) and HCT116 (3 × 10^5^ cells/well) cells were seeded in 6-well culture plates and allowed to adhere overnight. After 24 h of treatment with STL (0–20 μM), the cells were harvested and a 100 μL aliquot was mixed with Muse™ Annexin V & Dead Cell Reagent and incubated for 20 min in the dark. Apoptotic cells were quantified using a Muse^®^ Cell Analyzer. Changes in mitochondrial membrane potential were assessed using the Muse^®^ MitoPotential Kit according to the manufacturer’s instructions. Briefly, CT26 and HCT116 cells (1 × 10^5^ cells/well) were treated with STL (0–20 μM) for 24 h, harvested, and incubated with MitoPotential Dye and 7-AAD. Depolarized cells were quantified using a Muse^®^ Cell Analyzer.

### 2.5. Western Blot Analysis

Proteins were extracted from STL-treated cells and lung tissues using PRO-PREP™ protein extraction solution (iNtRon Biotechnology, Seoul, Republic of Korea). The lysates were vortexed every 10 min for 50 min and centrifuged at 13,000 rpm for 7 min at 4 °C to collect the supernatant. Protein samples were mixed with 4× SDS sample buffer and denatured at 95 °C for 5 min. Equal amounts of protein were separated by sodium dodecyl sulfate–polyacrylamide gel electrophoresis and transferred to polyvinylidene fluoride membranes. Membranes were blocked using EveryBlot Blocking Buffer (Bio-Rad Laboratories, Hercules, CA, USA) for 5 min prior to overnight incubation at 4 °C with the respective primary antibodies. After three washes with Tris-buffered saline containing 0.1% Tween 20 (TBST) over a total of 45 min, the membranes were incubated with the appropriate secondary antibodies for 60 min. Following another 45 min wash with 0.1% TBST, protein bands were detected using an enhanced chemiluminescence (ECL) solution and visualized with the Alliance Q9 Advanced imaging system (UVITEC, Cambridge, UK).

### 2.6. ROS Level Measurement

The Muse^®^ Oxidative Stress Kit (Luminex, Austin, TX, USA) was used to measure cellular ROS levels. The Muse^®^ Oxidative Stress Reagent and 1× Assay Buffer were equilibrated to room temperature. STL-treated cells (1 × 10^6^–1 × 10^7^ cells/mL) were prepared in 1× Assay Buffer. We transferred a 10 µL aliquot of the cell suspension into each tube and then added 190 µL of the Muse Oxidative Stress Reagent working solution. Samples were vortexed at medium speed for 5 s. The tubes were then loosely capped and incubated at 37 °C for 30 min. ROS levels were quantified using a Muse^®^ Cell Analyzer.

### 2.7. Total Antioxidant Capacity (TAC) Assay

TAC was measured using the EZ-Total Antioxidant Capacity Assay Kit (DG-TAC200; DoGenBio, Seoul, Republic of Korea) according to the manufacturer’s instructions. Briefly, cell lysates were mixed with 200 µL of working solution containing 100 µL of copper reagent and 100 µL of reaction buffer and incubated at room temperature for 30 min. Following the measurement of optical density at 450 nm using a microplate reader, the TAC was quantified in mM based on a Trolox calibration curve.

### 2.8. Measurement of Mitochondrial Superoxide Production

Cells treated with STL for 24 h were stained with MitoSOX™ Red Mitochondrial Superoxide Indicator for live-cell imaging (Thermo Fisher Scientific, Waltham, MA, USA) to detect mitochondrial superoxide generation. Stock and working solutions of the MitoSOX reagent were prepared according to the manufacturer’s instructions. For staining, the working solution was applied to cells adhering to coverslips in 35 mm dishes (1–2 mL per dish) or to cells in 96-well plates (100 µL per well), ensuring that the cell monolayers were completely submerged. Cells were maintained in the dark at 37 °C for 30 min within a humidified incubator containing 5% CO_2_. Subsequently, the samples were rinsed three times with prewarmed Hank’s Balanced Salt Solution. Stained cells were visualized within 2 h using an EVOS^®^ FL Cell Imaging System based on the reagent’s specific spectral properties.

### 2.9. Animal Experiment Using a Pulmonary Metastasis Model

Female BALB/c mice (5 weeks old, 17–18 g) were purchased from Samtako (Osan, Republic of Korea) and acclimated under controlled environmental conditions. The mice were maintained in a laminar-flow facility at 22 ± 1 °C and 55 ± 1% humidity under a 12 h light/dark cycle. Animals were group-housed (6 mice per cage) in individually ventilated cages lined with autoclaved aspen-chip bedding, with ad libitum access to standard laboratory chow and water. All in vivo procedures were conducted in strict accordance with the ARRIVE guidelines and the ethical standards approved by the Institutional Animal Care and Use Committee of Wonkwang University (WKU26-8). A total of 30 mice were randomly allocated to 5 experimental groups, with 6 animals per group (n = 6). As each individual animal was defined as the experimental unit, there were 6 experimental units per group, resulting in a total of 30 experimental units. The sample size (n = 6 per group) was determined based on previous literature employing similar disease models, which demonstrated sufficient statistical power to detect meaningful biological differences while adhering to the 3Rs principle to minimize animal use.

To establish the lung metastasis model, CT26 cells (2 × 10^5^ cells/100 μL) were administered by tail-vein injection. The mice were randomly assigned to five groups (n = 6 per group): (1) control; (2–4) STL-treated groups (12.5, 25, and 50 mg/kg); and (5) 5-fluorouracil (5-FU)-treated group (10 mg/kg). Animals were assigned to the experimental groups using stratified randomisation based on baseline body weights to ensure equivalent initial mean weights across all groups. The cage location was changed daily during drug administration. STL was administered orally once daily for 14 days, whereas 5-FU was administered intraperitoneally every 3 days during the same period. All drug administrations and outcome evaluations were conducted at a fixed time of day (between 10:00 and 11:30 AM) to eliminate circadian variations. After 2 weeks, the mice were humanely euthanized; serum and lung tissues were collected for biochemical analysis. To quantify metastatic nodules, the lungs were fixed and stained with Bouin’s solution. Trained personnel handled animals gently to minimize handling-induced stress, and provided a 7-day acclimatization period before the study began. Oral gavage was performed using smooth, stainless-steel ball-tipped feeding needles of appropriate gauge to prevent esophageal or mucosal injury. Restraint duration during administration was kept to a minimum.

To minimize experimental bias, an independent researcher assigned random identification numbers to the treatment solutions and animal cages, ensuring that the researchers administering the treatments remained unaware of this information. The same numbering system was consistently maintained during tissue collection and subsequent outcome assessment.

### 2.10. Statistical Analysis

All quantitative data are shown as the mean ± SD. Group comparisons were assessed using Student’s t-test alongside one-way ANOVA, followed by Tukey’s post hoc test. IBM SPSS Statistics (v26, IBM Corp., Armonk, NY, USA) served as the tool for all statistical processing, with significance established at * *p* < 0.05, ** *p* < 0.01, and *** *p* < 0.001.

## 3. Results

### 3.1. STL Inhibits the Proliferation and Clonogenic Potential of CRC Cells

**Figure 1:**
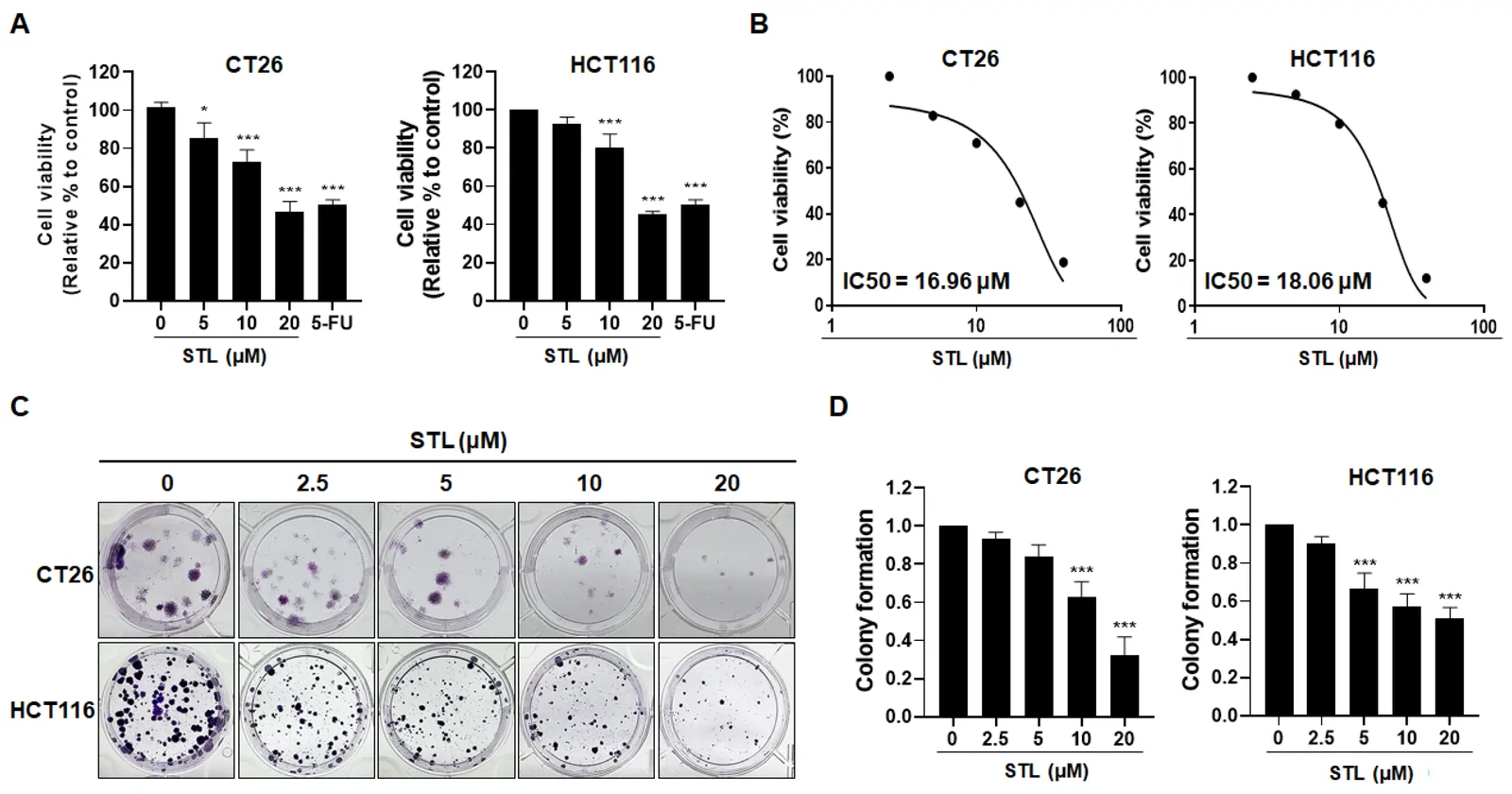
STL inhibits the viability and colony-forming ability of CRC cells. (**A**) Viability of CT26 and HCT116 cells after 24 h of STL treatment. 5-FU (10 μM) was treated to CRC cells for 24 h as a positive control. (**B**) IC50 values of STL in CT26 and HCT116 cells. (**C**) Colony formation by CT26 and HCT116 cells after 1 week of STL treatment. Representative images from each group are shown. (**D**) Normalized colony formation rate. Data are presented as the mean ± SD from three independent experiments. * *p* < 0.05 and *** *p* < 0.001.

The impact of STL on cellular viability was measured in CT26 and HCT116 cells treated across a 0–40 μM concentration gradient for 24 h, utilizing the CCK reagent for quantification. In both cell lines, STL reduced cell viability in a concentration-dependent manner at concentrations of 10 μM or higher (Figure 1A). Treatment with 10 μM 5-FU for 24 h resulted in cell viability levels in both CT26 and HCT116 cells comparable to those observed with 20 μM STL. The IC50 values of STL in CT26 and HCT116 cells were 16.96 and 18.06 μM, respectively (Figure 1B). Furthermore, a clonogenic assay was conducted to evaluate the colony-forming potential of CRC cells. Colony formation decreased in a concentration-dependent manner after STL treatment (Figure 1C,D). To investigate whether the cytotoxic effect of STL is tumor-selective, we evaluated cell viability in the normal colon fibroblast cell line CCD-18Co and the mast cell line RBL-2H3 under equivalent STL concentrations. Both CCD-18Co and RBL-2H3 cells maintained over 80% viability following treatment with 10 μM STL and retained greater than 70% viability even at 20 μM (Supplementary Figure S1). Collectively, these results indicate that concentrations of STL cytotoxic to colorectal cancer cells exhibit relatively minimal cytotoxicity toward non-malignant cells, demonstrating its selective antitumor potential.

### 3.2. STL Induces Caspase-Mediated Apoptosis in CRC Cells

**Figure 2:**
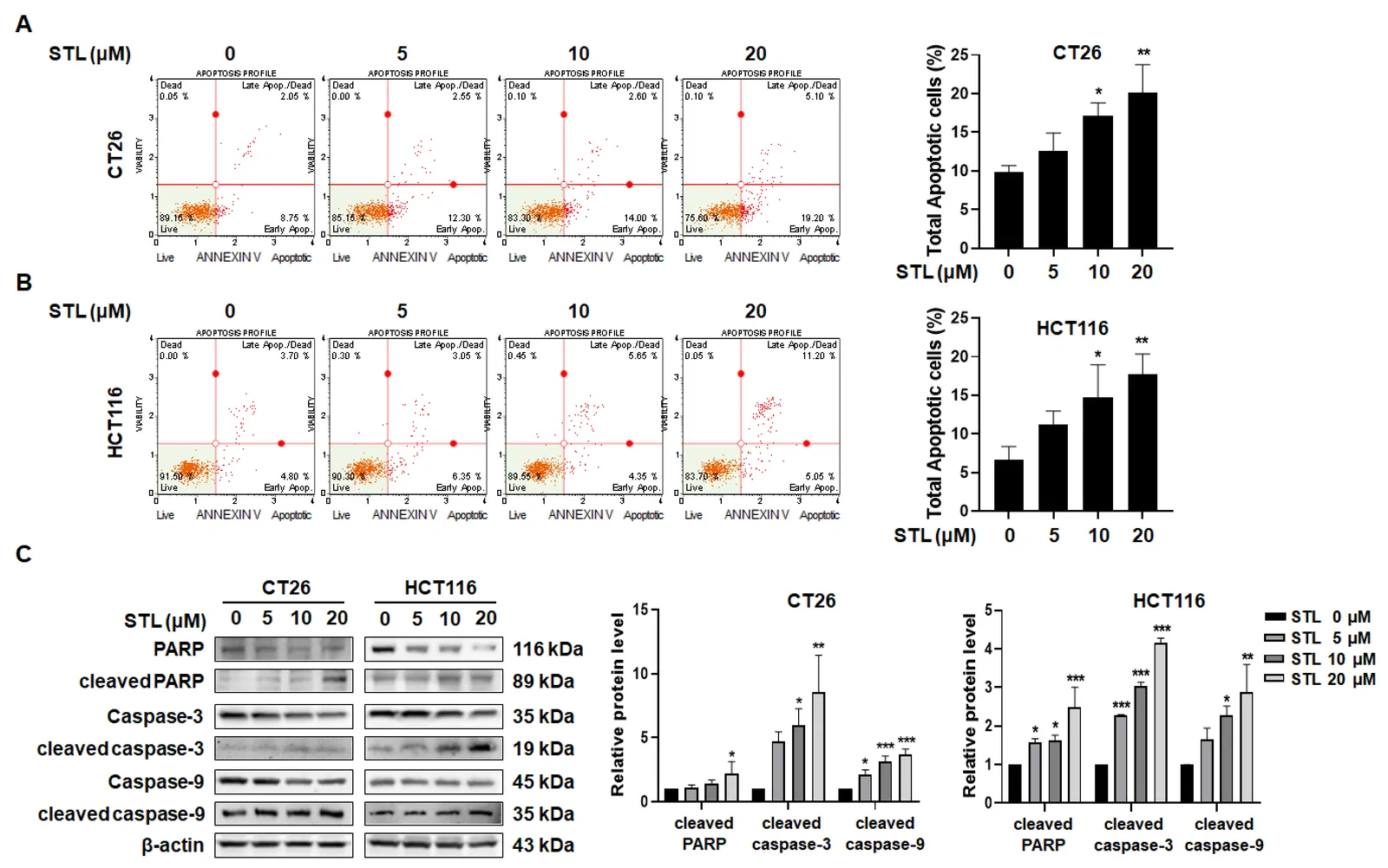
STL increases caspase-mediated apoptosis in CRC cells. (**A**,**B**) Annexin V staining of CT26 (**A**) and HCT116 (**B**) cells after 24 h of STL treatment. The cells were analyzed by flow cytometry. Red solid lines indicate the boundaries between live, early/late apoptotic, and dead cell populations. The graphs show the percentages of total apoptotic cells after STL treatment. Representative images from each group are shown. (**C**) Expression of apoptosis-related proteins (**left**) and the corresponding relative protein levels (**right**) in STL-treated cells. Data are presented as the mean ± SD from three independent experiments. * *p* < 0.05, ** *p* < 0.01, and *** *p* < 0.001.

To investigate the proapoptotic effect of STL on CRC cells, we performed an Annexin V assay using flow cytometry. STL induced concentration-dependent apoptosis in both CT26 and HCT116 cells. A significant increase in apoptosis was observed at STL concentrations greater than 10 μM (Figure 2A,B). To further investigate the underlying mechanism, Western blotting was performed and showed that STL treatment induced cleavage of caspase-3, caspase-9, and PARP (Figure 2C). These findings indicate that STL promotes cell death through a caspase-dependent apoptotic pathway.

### 3.3. STL-Induced Apoptosis Is Mediated by Loss of Mitochondrial Membrane Potential in CRC Cells

**Figure 3:**
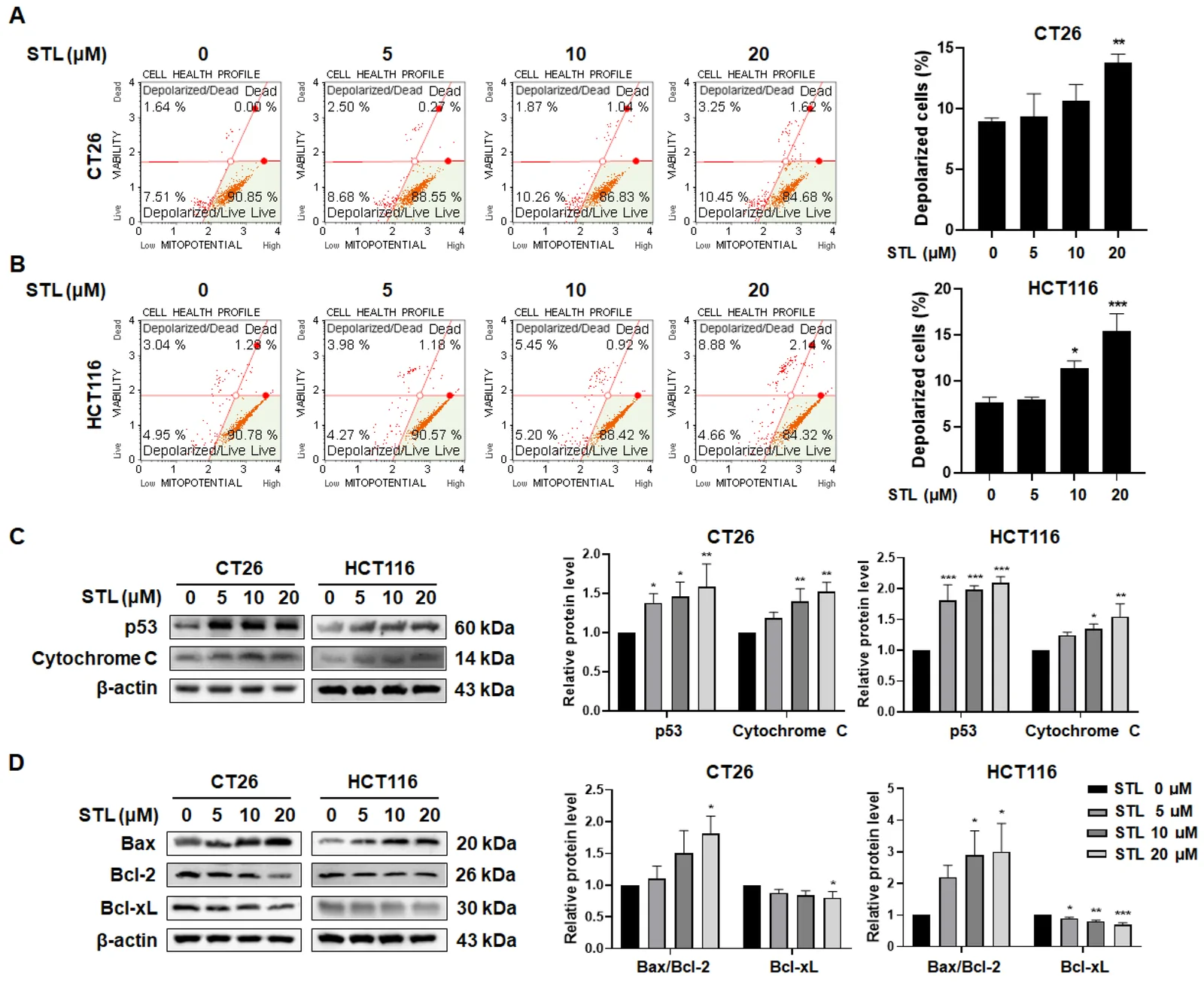
STL triggers apoptosis through mitochondrial membrane depolarization in CRC cells. (**A**,**B**) Flow cytometric analysis of mitochondrial depolarization in CT26 (**A**) and HCT116 (**B**) cells after 24 h of STL exposure. Red solid lines delineate viable, depolarized/viable, depolarized/necrotic, and necrotic cell populations. The graphs show the percentages of cells exhibiting mitochondrial depolarization after STL treatment. Representative images from each group are shown. (**C**,**D**) Expression and densitometric quantification of p53, cytochrome c, Bax, Bcl-2, and Bcl-xL in STL-treated CT26 and HCT116 cells. Protein levels were normalized to β-actin as the loading control. Data are presented as the mean ± SD from three independent experiments. * *p* < 0.05, ** *p* < 0.01, and *** *p* < 0.001.

Because STL induced caspase-9 cleavage, we examined mitochondrial membrane potential to determine whether STL-induced apoptosis in CRC cells was mediated by the intrinsic mitochondrial pathway. After 24 h of STL treatment, mitochondrial depolarization increased significantly in both CT26 and HCT116 cells (Figure 3A,B). To further elucidate the mechanism of STL-induced CRC cell death, Western blotting was used to analyze p53, a key regulator of apoptosis, and proteins associated with the intrinsic pathway. STL treatment increased cytochrome c release into the cytosol, consistent with increased mitochondrial outer membrane permeability, and significantly increased p53 expression (Figure 3C). Among downstream Bcl-2 family proteins, the expression of proapoptotic Bax increased significantly, whereas the expression of antiapoptotic Bcl-2 and Bcl-xL decreased by STL treatment (Figure 3D). These findings indicate that STL alters mitochondrial membrane potential and induces apoptosis in CRC cells through the intrinsic pathway.

### 3.4. STL Increases Mitochondria-Derived ROS Production

**Figure 4:**
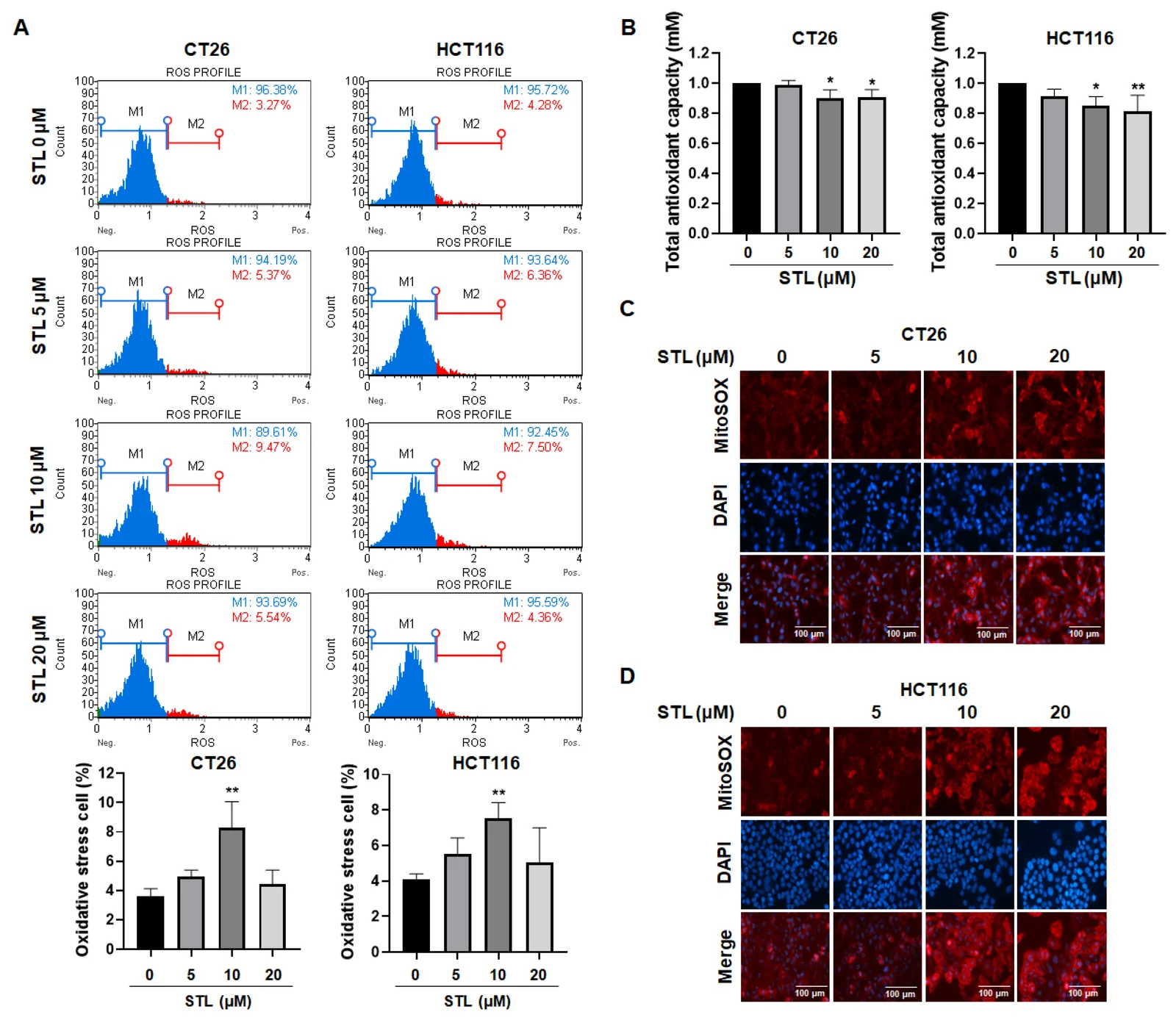
STL induces mitochondrial ROS production in CT26 and HCT116 cells. Cells were treated with various concentrations of STL (0–20 μM) for 24 h. (**A**) Flow cytometric analysis of cellular ROS levels using the Muse^®^ Oxidative Stress Kit, which distinguishes ROS^−^ (M1) and ROS^+^ (M2) populations. The bar graph shows the percentage of M2 cells after STL treatment. (**B**) TAC levels in STL-treated CT26 and HCT116 cells. (**C**,**D**) Representative fluorescence images of MitoSOX-stained CT26 (**C**) and HCT116 (**D**) cells. Data are presented as the mean ± SD from three independent experiments. * *p* < 0.05, and ** *p* < 0.01.

To determine whether STL induces mitochondrial ROS production in CRC cells, flow cytometry was also used to assess ROS production. STL treatment significantly increased the percentage of ROS-positive CRC cells (Figure 4A). At the highest tested concentration of STL (20 μM), DCF fluorescence intensity decreased compared to the intermediate dose; this appears to be attributable to extensive cell death and the loss of membrane integrity associated with high-concentration treatment. Figure 4B shows the total antioxidant capacity of CT26 and HCT116 cells treated with various concentrations of STL (0–20 μM). In both cell lines, total antioxidant capacity decreased in a concentration-dependent manner as the STL concentration increased. In addition, CT26 and HCT116 cells were treated with STL for 24 h and then stained with MitoSOX. STL concentrations of 10 μM or higher significantly increased MitoSOX fluorescence intensity in both cell lines, indicating increased mitochondrial superoxide generation (Figure 4C,D). These findings suggest that STL impairs antioxidant defenses and increases ROS production in CRC cells, thereby reducing their total antioxidant capacity. Additionally, we investigated whether NAC, a specific ROS scavenger, could reverse STL-mediated cytotoxicity. Following the confirmation of NAC activity (Supplementary Figure S2A), CT26 and HCT116 cells were pretreated with NAC for 1 h before STL administration. As a result, NAC pretreatment effectively rescued cell viability from STL-induced cytotoxicity in both cell lines (Supplementary Figure S2B). These findings indicate that STL increases apoptosis in CRC cells by inducing excessive ROS production.

### 3.5. STL Regulates PI3K/AKT, AMPK, and MAPK Signaling Pathways in CRC Cells

**Figure 5:**
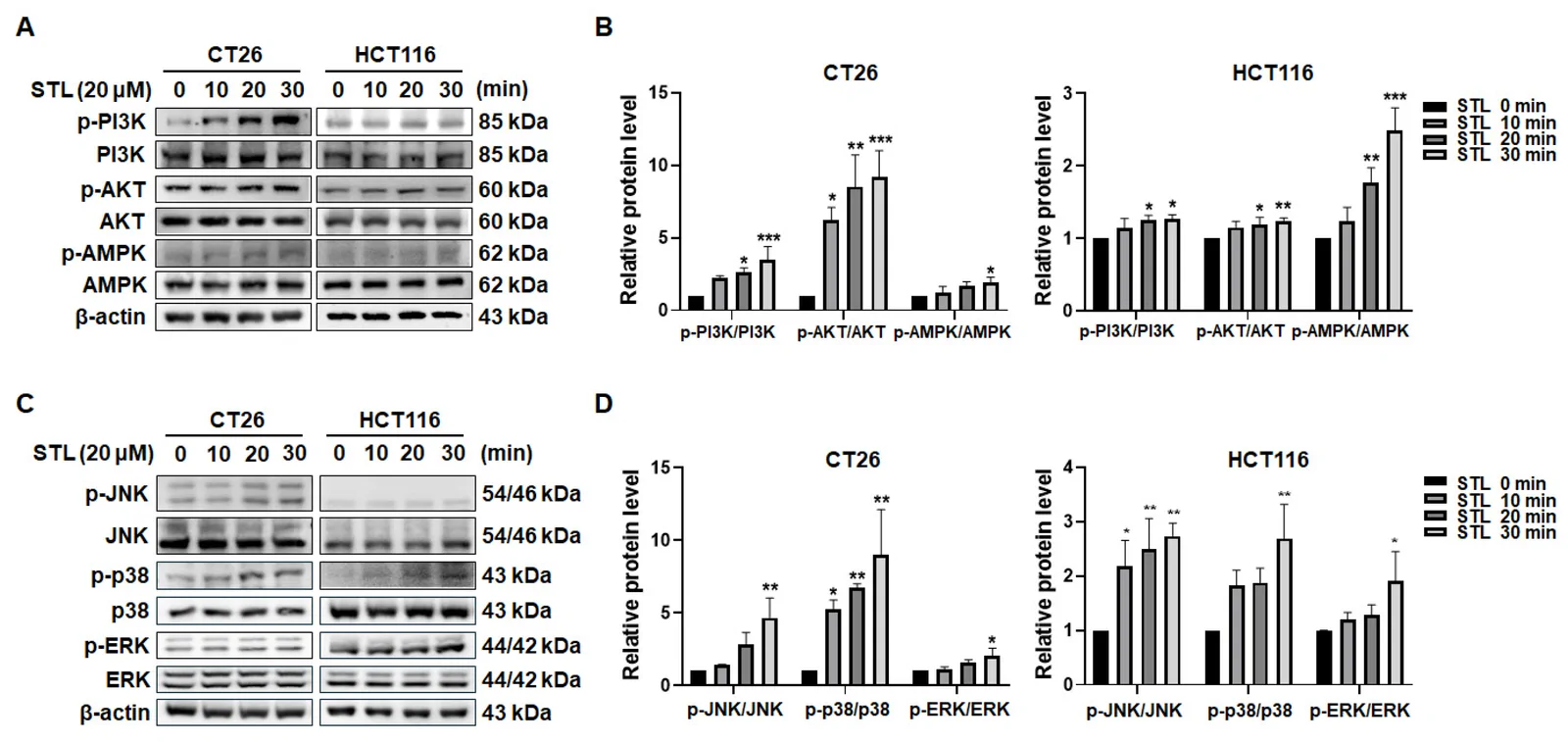
STL regulates apoptosis-related signaling pathways. (**A**) PI3K/AKT and AMPK phosphorylation in STL-treated CT26 and HCT116 cells. (**B**) Densitometric quantification of protein bands using ImageJ. (**C**) Phosphorylation of JNK, p38, and ERK in CRC cells after STL treatment. (**D**) Densitometric quantification of the protein bands using ImageJ. Representative images from each group are shown. Data are presented as the mean ± SD from three independent experiments. * *p* < 0.05, ** *p* < 0.01, and *** *p* < 0.001.

Reactive oxygen species (ROS) contribute to cancer progression by promoting tumor cell survival and proliferation, thereby facilitating tumor growth and progression [28]. To identify signaling factors regulated by STL during ROS-associated apoptosis in CRC cells, we examined the phosphorylation of several signaling proteins by Western blotting. As shown in Figure 5A, short-term (10–30 min) STL treatment increased the phosphorylation of PI3K and AKT and increased AMPK phosphorylation in a concentration-dependent manner. Densitometric analysis using ImageJ software (version 1.54g) showed that the ratios of phosphorylated to total proteins increased significantly at higher STL concentrations in both cell lines (Figure 5B). We also examined the MAPK signaling pathway, which is involved in cell survival and apoptosis. STL significantly increased JNK, p38, and ERK phosphorylation in CT26 and HCT116 cells (Figure 5C,D). Additionally, we examined the effects of AMPK and MAPK inhibition on cell survival following STL exposure. Pretreatment with the AMPK inhibitor Compound C significantly increased cell viability relative to treatment with STL alone (Supplementary Figure S3). These results indicate that the cytotoxic effect of STL against CRC cells is mediated by AMPK.

### 3.6. STL Reduces Lung Metastasis of CRC Cells by Inducing Apoptosis in a Mouse Model

**Figure 6:**
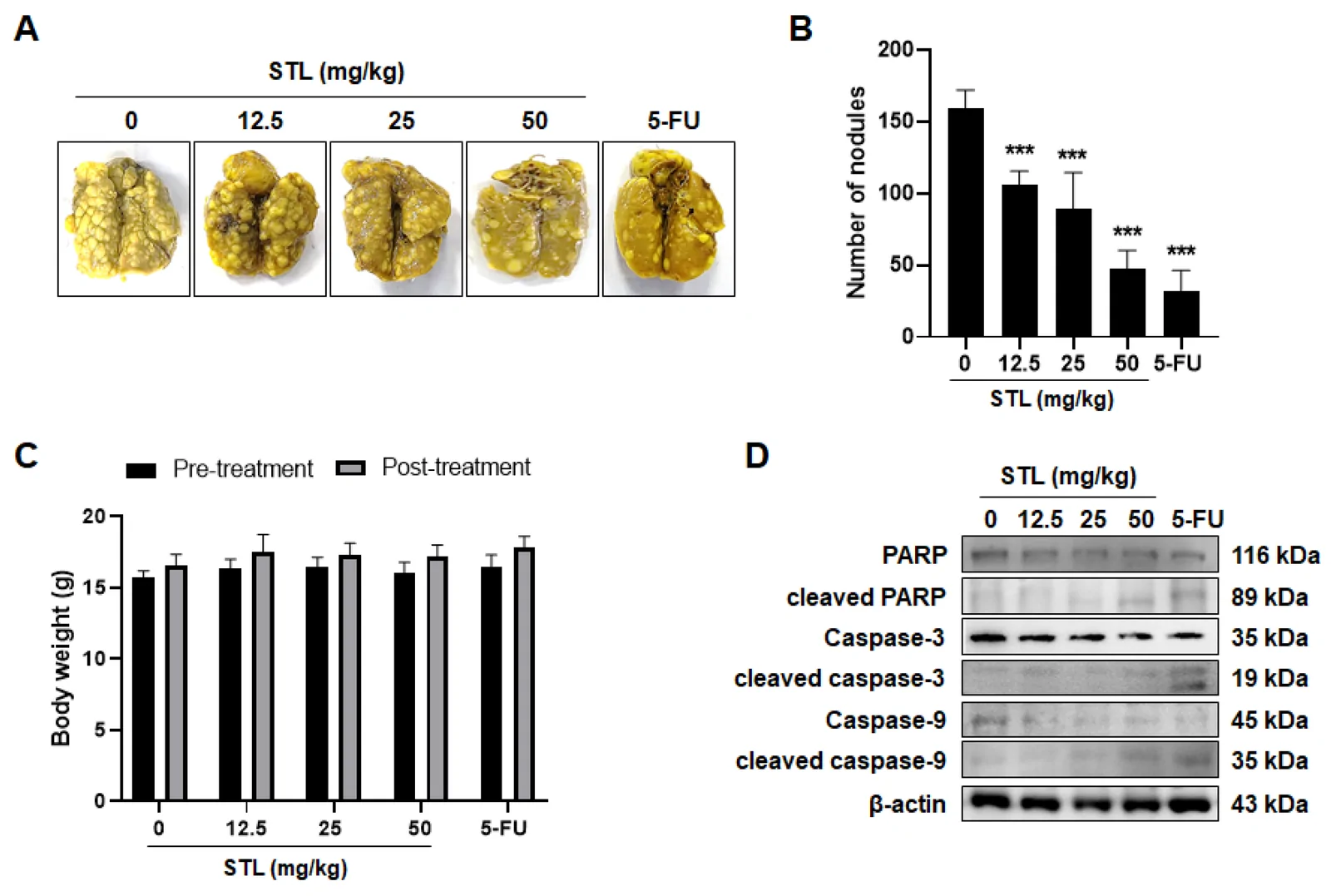
STL suppresses lung metastasis of CT26 cells by inducing apoptosis in a mouse model. (**A**) Representative images of lung tissues collected 14 days after the in vivo experiment and fixed and stained with Bouin’s solution. (**B**) Total number of metastatic lung nodules. (**C**) Body weight of STL-treated mice. (**D**) Expression of proteins associated with apoptotic cell death in the lungs after 14 days of oral STL administration. Representative images are shown, and data are presented as the mean ± SD. *** *p* < 0.001.

To determine whether STL suppresses lung metastasis by inducing apoptosis in CRC cells, we established a pulmonary colonization model established by tail-vein injection and conducted in vivo experiments. After 2 weeks of oral STL administration, dose-dependent reduction in the formation of CT26-derived metastatic lung nodules was observed (Figure 6A). STL at 25 and 50 mg/kg reduced the number of nodules to approximately 55% and 30% of the control value, respectively (Figure 6B). Administration of 10 mg/kg 5-FU led to an average additional reduction of approximately 10% in macroscopic lung surface nodules compared to 50 mg/kg STL. Body weight was measured at the beginning of the experiment (Day 1) and on the day of euthanasia (Day 14) to monitor potential toxicity. No treatment-related changes in body weight were observed during the experimental period (Figure 6C). These findings suggest that oral STL at doses up to 50 mg/kg did not cause overt systemic toxicity during the experimental period. Western blot analysis of lung tissue showed that STL treatment increased cleavage of caspase-3, caspase-9, and PARP (Figure 6D). In addition, serum markers of potential hepatic and renal toxicity did not change significantly after STL administration (Table 1). These findings were consistent with those of the in vitro experiments. Collectively, the results demonstrate that STL suppresses lung metastasis by inducing apoptosis in CRC cells and confirm its efficacy in vivo.

## 4. Discussion

In this study, we demonstrated the potential of STL for CRC treatment by showing that it induces excessive ROS generation and subsequent apoptosis in CRC cells. STL is an abundant bioactive compound in sandalwood; both sandalwood oil and STL have been reported to exert therapeutic effects against several cancer types [23,24,25,26,29]. An ethanolic sandalwood extract selectively induced apoptosis in A375 and SK-MEL-3 human melanoma cells by significantly increasing ROS formation and triggering cytochrome c release [30]. Previous studies have also shown that STL (25–75 μM) induces apoptosis in human epidermoid carcinoma A431 cells through caspase activation mediated by loss of mitochondrial membrane potential and cytochrome c release [24]. STL treatment induces apoptosis in human prostate cancer cells (PC-3 and LNCaP) through caspase-3 and PARP cleavage [25] and in human breast cancer cells (MCF-7 and MDA-MB-231) through cleavage of caspase-9, caspase-3, and PARP [26]. In non-small cell lung cancer cells, STL inhibits the viability of A549 and PC9 cells by regulating Bax and Bcl-2 expression [27]. In our experiments, we first determined the changes in CRC cell viability following treatment with graded concentrations of STL, and confirmed that STL exhibited antitumor efficacy and anti-colonization activity at concentrations that preserved high viability (>70–80%) in normal colon fibroblast (CCD-18Co) and mast cells (RBL-2H3). STL (5–20 μM) increased ROS levels and apoptosis in CT26 and HCT116 cells. This response was mediated by regulation of Bcl-2, Bcl-xL, and Bax expression, leading to cytochrome c release and subsequent cleavage of caspase-9, caspase-3, and PARP. Consequently, the accumulation of reactive oxygen species (ROS) triggered by STL disrupts the mitochondrial membrane potential, facilitating the translocation of proapoptotic molecules like cytochrome c from the mitochondria to the cytosol. The released cytochrome c facilitates apoptosome formation and activation of the caspase-9/3 pathway, ultimately inducing apoptosis.

Natural compounds frequently induce cancer cell apoptosis via ROS-mediated mechanisms across diverse signaling networks [31]. It is well-documented that PI3K/AKT signaling and cellular redox status share a reciprocal relationship. Specifically, PI3K/AKT pathway activation upregulates ROS production through mitochondrial pathways and NADPH oxidases, whereas elevated ROS reciprocally hyperactivates PI3K/AKT signaling by suppressing negative regulators such as PTEN via oxidation. This feedback loop forms a crucial mechanism that supports tumor proliferation, survival, and drug resistance [32]. In addition, excessive ROS activates AMPK and MAPK signaling pathways, including JNK, p38, and sustained ERK signaling, leading to mitochondrial dysfunction and caspase-dependent apoptosis in CRC cells. Unlike transient ERK activation, which is associated with cell proliferation, sustained ROS-mediated ERK activation functions as a proapoptotic signal. Accordingly, various pharmacological agents and natural compounds induce apoptosis through ROS-dependent activation of AMPK/MAPK signaling, highlighting these pathways as key mediators of ROS-induced CRC cell death [33,34]. For example, ampelopsin, a natural product-derived compound, promotes apoptosis in several human-derived CRC cell lines by inducing ROS generation and activating the endoplasmic reticulum stress-mediated AMPK/MAPK/XAF1 signaling pathway [35]. In the present study, STL increased PI3K/AKT, AMPK, and MAPK phosphorylation in CRC cells. When STL activates PI3K/AKT and generates excessive ROS, cells experience severe oxidative and metabolic stress. The concurrent activation of AMPK, JNK, p38, and ERK may represent a response to ROS-induced stress and contribute to the execution of apoptosis. Specifically, assessing the rescue effects of these signaling pathway inhibitors revealed that pretreatment with the AMPK inhibitor Compound C markedly attenuated the STL-induced reduction in cell viability.

Reactive oxygen species (ROS) promote cancer metastasis by inducing epigenetic remodeling and transcriptional changes that enhance epithelial–mesenchymal transition, invasion, and migration of cancer cells [36]. To determine whether the proapoptotic effect of STL on CRC cells also suppresses lung metastasis, we used a mouse lung metastasis model established by intravenous injection. Previous studies showed that STL (100 mg/kg) exerted antioxidant effects when administered intraperitoneally for 2 weeks to mice with D-galactose-induced oxidative stress and antihyperglycemic effects when administered intraperitoneally for 1 week to mice with alloxan-induced diabetes [37]. Another study reported that administering STL (30–70 mg/kg) to mice for 2 weeks increased brain levels of homovanillic acid, 3,4-dihydroxyphenylacetic acid, and 5-hydroxyindoleacetic acid and altered central nervous system activity [38]. Because STL is hydrophobic and sparingly soluble in water, it was dissolved in DMSO for in vitro assays. For the in vivo experiments, STL was formulated as a suspension in distilled water; however, considering the physical limits of syringeability and smooth delivery through an oral gavage needle without precipitation or blockage, 50 mg/kg represented the highest feasible dose for oral administration in mice. Based on these findings, we considered oral administration of STL at doses of 12.5–50 mg/kg for 2 weeks to be safe and proceeded with the in vivo experiments. Serum markers related to hepatic and renal function did not change significantly in the STL-treated groups. These findings suggest that STL may induce apoptosis in CRC cells and inhibit distant metastasis at the tested doses. Pulmonary metastasis is one of the most lethal complications in patients with advanced CRC. The current study demonstrates that STL significantly reduces metastatic tumor burden in a murine tail-vein injection model, suggesting its potential translational value in managing circulating tumor cell colonization in the lungs. However, several translational considerations remain. Further investigations utilizing orthotopic models and comprehensive pharmacokinetic profiles will be necessary to substantiate the generalisability of these findings across broader preclinical and clinical settings.

## 5. Conclusions

Our findings demonstrate that STL suppresses the proliferation and metastatic potential of CRC cells. This effect appears to result from STL-induced oxidative stress, characterized by reduced total intracellular antioxidant capacity and increased ROS generation. This process is associated with mitochondria-mediated apoptosis and activation of the PI3K/AKT, AMPK, and MAPK signaling pathways. These findings identify STL as a promising candidate for the development of novel CRC therapies.

## Figures and Tables

**Table 1:** Serum levels of AST, ALT, creatinine, and BUN in STL-treated mice.

	STL 0 mg/kg	STL 12.5 mg/kg	STL 25 mg/kg	STL 50 mg/kg	5-FU 10 mg/kg
AST (IU/L)	120.57 ± 45.90	132.66 ± 35.57	125.5 ± 44.13	97.00 ± 14.73	99.27 ± 36.73
ALT (IU/L)	23.50 ± 4.18	25.25 ± 3.46	26.25 ± 2.44	21.66 ± 3.01	21.90 ± 5.04
Creatinine (mg/dL)	0.21 ± 0.03	0.20 ± 0.03	0.20 ± 0.03	0.21 ± 0.04	0.17 ± 0.03
BUN (mg/dL)	25.90 ± 5.54	23.00 ± 3.45	22.6 ± 2.04	23.00 ± 1.15	26.27 ± 3.71

## Data Availability

The original contributions presented in the study are included in the article; further inquiries can be directed to the corresponding authors.

## Supplementary Materials

The following supporting information can be downloaded at: https://www.mdpi.com/article/10.3390/antiox15101361/s1, Figure S1: Effects of STL on cell viability in CCD-18Co and RBL-2H3 cells; Figure S2: STL-induced cytotoxicity and reduction in total antioxidant capacity were attenuated by N-acetylcysteine (NAC) in CRC cells; Figure S3: Inhibitory effect of Compound C (CC) on STL-induced cytotoxicity in CRC cells.
