# Supplementary material for: Santalol Suppresses Lung Metastasis of Colorectal Cancer Cells by Increasing ROS Levels
Source: Antioxidants (Basel). 2026 Oct 21;15(10):1361. doi: 10.3390/antiox15101361 (PMC13701331; doi:10.3390/antiox15101361)
Supplement: Supplementary file 1 [file antioxidants-15-01361-s001.zip › antioxidants-4544406-supplementary.pdf]

Supplementary Figure

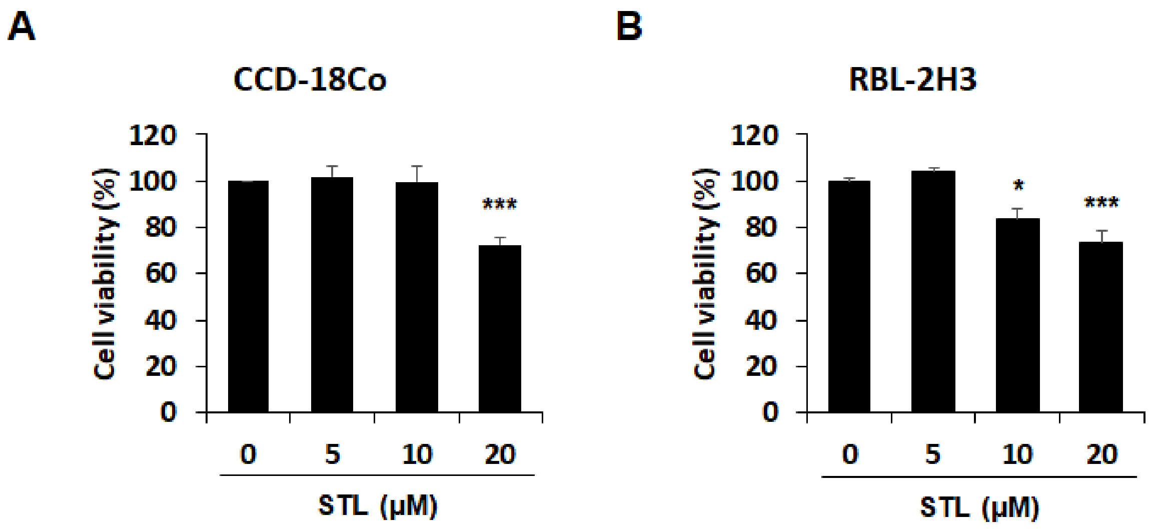

Figure S1. Effects of STL on cell viability in CCD-18Co and RBL-2H3 cells. CCD-18Co and RBL-2H3 cells were seeded at 1 × 10^4 cells/well in 96-well plates and incubated overnight. Cells were treated with STL (5, 10, and 20 μM) for 24 h, followed by cell viability measurement using a CCK reagent. Data are presented as the mean ± SD of three independent experiments.*$p < 0.05$ and ***$p < 0.001$.

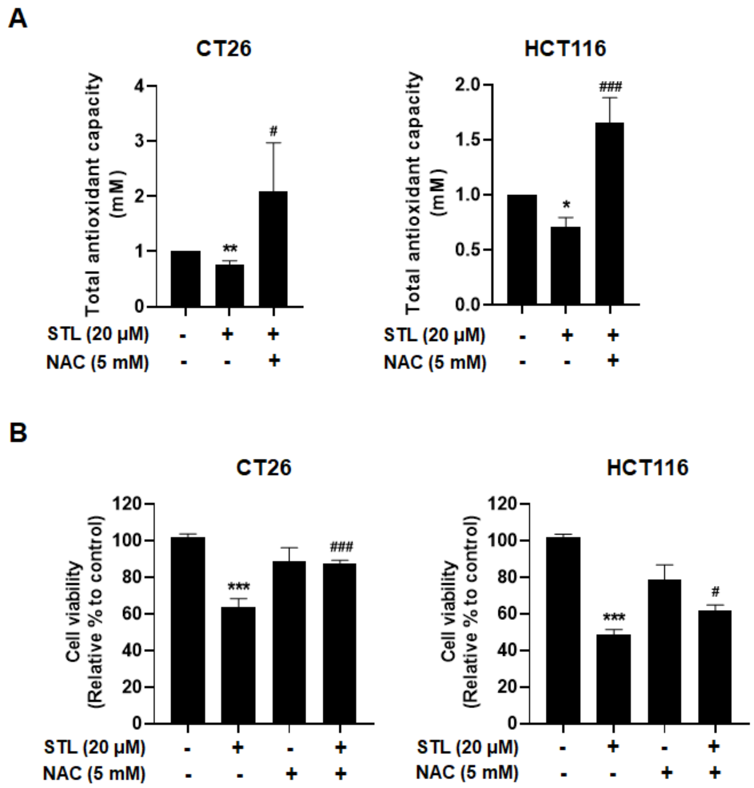

Figure S2. STL-induced cytotoxicity and reduction in total antioxidant capacity were attenuated by N-acetylcysteine (NAC) in CRC cells. (A) TAC in CT26 and HCT116 cells treated with STL alone or co-treated with NAC. (B) Cell viability of CT26 and HCT116 cells treated with STL in the presence or absence of NAC. Cells were pretreated with 5 mM NAC for 1 h prior to treatment with 20 μM STL for 24 h. Data are expressed as mean ± SD of independent experiments. *$p < 0.05$, **$p < 0.01$, and ***$p < 0.001$ vs. the control group; #$p < 0.05$ and ###$p < 0.001$ vs. the STL-treated group.

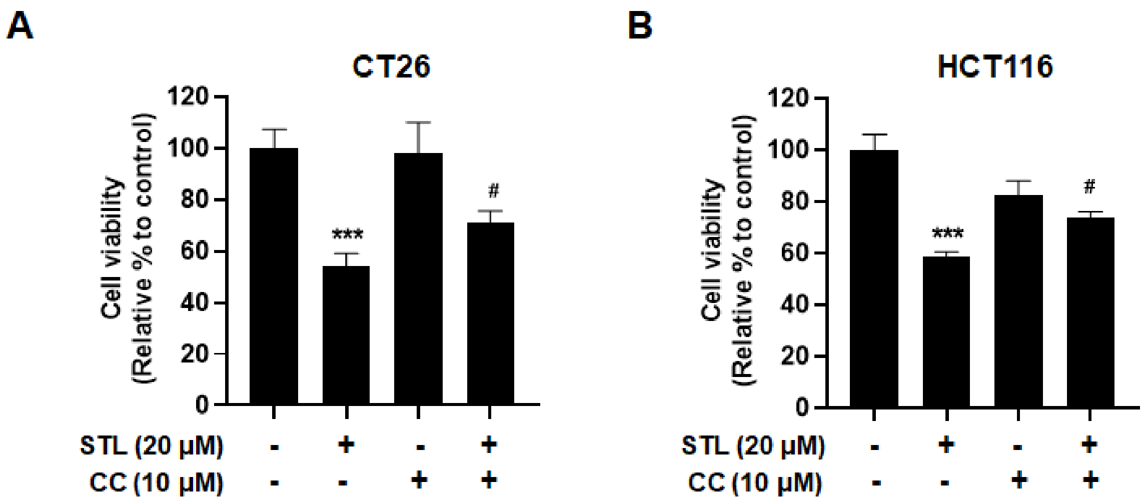

Figure S3. Inhibitory effect of Compound C (CC) on STL-induced cytotoxicity in CRC cells. (A and B) Cell viability of STL-treated CT26 (A) and HCT116 (B) cells after CC treatment. Cells were pretreated with 10 μM CC for 1 h prior to treatment with 20 μM STL for 24 h. Data are expressed as mean ± SD of independent experiments. ***$p < 0.001$ vs. the control group; #$p < 0.05$ vs. the STL-treated group.
